# Genetics, Genomics and Evolution of Ergot Alkaloid Diversity

**DOI:** 10.3390/toxins7041273

**Published:** 2015-04-14

**Authors:** Carolyn A. Young, Christopher L. Schardl, Daniel G. Panaccione, Simona Florea, Johanna E. Takach, Nikki D. Charlton, Neil Moore, Jennifer S. Webb, Jolanta Jaromczyk

**Affiliations:** 1 Forage Improvement Division, The Samuel Roberts Noble Foundation, Ardmore, OK 73401, USA; E-Mails: jtakach@greenandgrow.com (J.E.T.); ndcharlton@noble.org (N.D.C.); 2 Department of Plant Pathology, University of Kentucky, Lexington, KY 40546, USA; E-Mails: schardl@uky.edu (C.L.S.); sflor2@uky.edu (S.F.); 3 Division of Plant and Soil Sciences, West Virginia University, Morgantown, WV 26506, USA; E-Mail: danpan@wvu.edu; 4 Computer Science Department, University of Kentucky, Lexington, KY 40546, USA; E-Mail: neil@uky.edu; 5 Advanced Genetic Technologies Center, University of Kentucky, Lexington, KY 40546, USA; E-Mails: jswebb2@uky.edu (J.S.W.); jolanta.jaromczyk@uky.edu (J.J.)

**Keywords:** *Claviceps*, *Epichloë*, *Periglandula*, Clavicipitaceae, gene clusters, chanoclavine, ergopeptine, subterminal, natural products, secondary metabolism

## Abstract

The ergot alkaloid biosynthesis system has become an excellent model to study evolutionary diversification of specialized (secondary) metabolites. This is a very diverse class of alkaloids with various neurotropic activities, produced by fungi in several orders of the phylum Ascomycota, including plant pathogens and protective plant symbionts in the family Clavicipitaceae. Results of comparative genomics and phylogenomic analyses reveal multiple examples of three evolutionary processes that have generated ergot-alkaloid diversity: gene gains, gene losses, and gene sequence changes that have led to altered substrates or product specificities of the enzymes that they encode (neofunctionalization). The chromosome ends appear to be particularly effective engines for gene gains, losses and rearrangements, but not necessarily for neofunctionalization. Changes in gene expression could lead to accumulation of various pathway intermediates and affect levels of different ergot alkaloids. Genetic alterations associated with interspecific hybrids of *Epichloë* species suggest that such variation is also selectively favored. The huge structural diversity of ergot alkaloids probably represents adaptations to a wide variety of ecological situations by affecting the biological spectra and mechanisms of defense against herbivores, as evidenced by the diverse pharmacological effects of ergot alkaloids used in medicine.

## 1. Importance of Ergot Alkaloids

Ergot alkaloids are well known mycotoxins that can contaminate food and feed but also can serve as starting materials for important pharmaceuticals. The ergot fungi, for which these alkaloids are named, have been responsible for historic episodes of mass poisoning. In Middle-Age Europe, ingestion of rye grain or flour that was contaminated with ergots—the resting stage (sclerotia) of *Claviceps purpurea*—led to multiple episodes of disfiguring and deadly poisoning of local populations. Historic events associated with ergot poisoning include the first Crusade [1], the Salem witch trials (and others) [2,3,4], and the interrupted 1722 Russian campaign (under Peter the Great) against the Ottoman empire [5]. In modern times, ergot-alkaloid poisoning occasionally occurs through their medicinal use [6], but mass ergot poisonings of humans are rare, having last been reported in the 1970s [7,8]. Ergot alkaloid toxicity is still a significant problem with livestock, both due to ergot contaminated feed and naturally infested forage or rangeland grasses—such as tall fescue (*Lolium arundinaceum*), sleepygrass (*Achnatherum robustum*) and drunken horse grass (*Achnatherum inebrians*)—which can be symbiotic with seed-transmissible *Epichloë* species that are capable of producing ergot alkaloids [9,10,11].

Over the past two decades, ergot alkaloids have been tapped for increasingly diverse medical uses. The ergopeptine ergotamine is used to treat migraines [12], and other natural and semisynthetic ergopeptines and dihydroergopeptines have been used for diseases of the brain. For example, bromocriptine (2-bromo-ergocryptine) is used as a component in treatment of Parkinsonism [13] and bromocriptine, or the extensively substituted dihydrolysergic acid amide, cabergoline, is used in treatment of prolactinoma, a benign adenoma of the pituitary gland [14,15]. Ergot alkaloids are also of social relevance because a semisynthetic alkaloid, lysergic acid diethylamide (LSD), is an illicit drug that is by far the most potent hallucinogen known. LSD had a major impact on the countercultural and hippie movements of the 1960s, since Albert Hofmann first produced it and noted its properties [16].

## 2. Structural Content of Ergot Alkaloid Biosynthesis (*EAS*) Loci Define Alkaloid Potential

**Figure 1:**
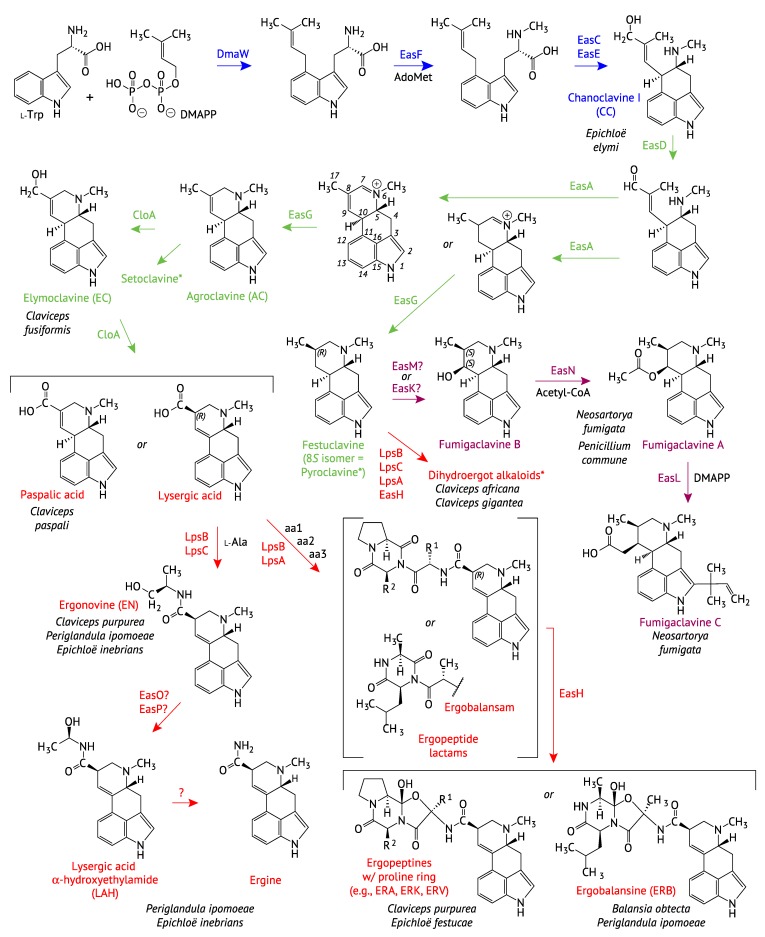
The ergot alkaloid pathway showing steps that result in diversification of compounds. Pathway steps are color-coded based on the position or diversification within the pathway, Blue = early steps to the intermediate chanoclavine, Green = mid steps leading to the tetracyclic clavines, Red = late steps represented by the lysergic acid amides and the complex ergopeptines, Purple = steps to fumigaclavines produced by Trichocomaceae.

The ergot alkaloids represent a diverse class of natural products that are generally divided into three subclasses: the simpler clavines, lysergic acid and its simple amides, and the highly complex ergopeptines. Figure 1 shows the simpler clavines in blue, green, and purple, and the lysergic acid and simple amides and ergopeptines both in red, reflective of the orders of biosynthetic steps: blue for early, green for middle, and purple and red for late steps in different fungal families. Clavines are tricyclic or tetracyclic compounds known from the fungal families Clavicipitaceae (order Hypocreales) and Trichocomaceae (Eurotiales), with the latter showing more variation due to hydroxylation, prenylation and acetylation (reviewed in [17]). The presence of *EAS* clusters in the Arthrodermataceae (Onygenales) suggests that they also may produce clavines, a possibility that is supported by the demonstration of chanoclavine I dehydrogenase activity of the *Arthroderma benhamiae* EasD ortholog (GenBank accession EFE37118.1) [18]. Lysergic acid and the lysergic acid amides are tetracyclic compounds with a common ergolene core, whereas ergopeptines are lysergic acid-tripeptide derivatives. These more complex ergot alkaloids are almost exclusively known from Clavicipitaceae, although an ergopeptine has also been reported from a *Dicyma* sp. (Xylariaceae, Xylariales) [19].

**Figure 2:**
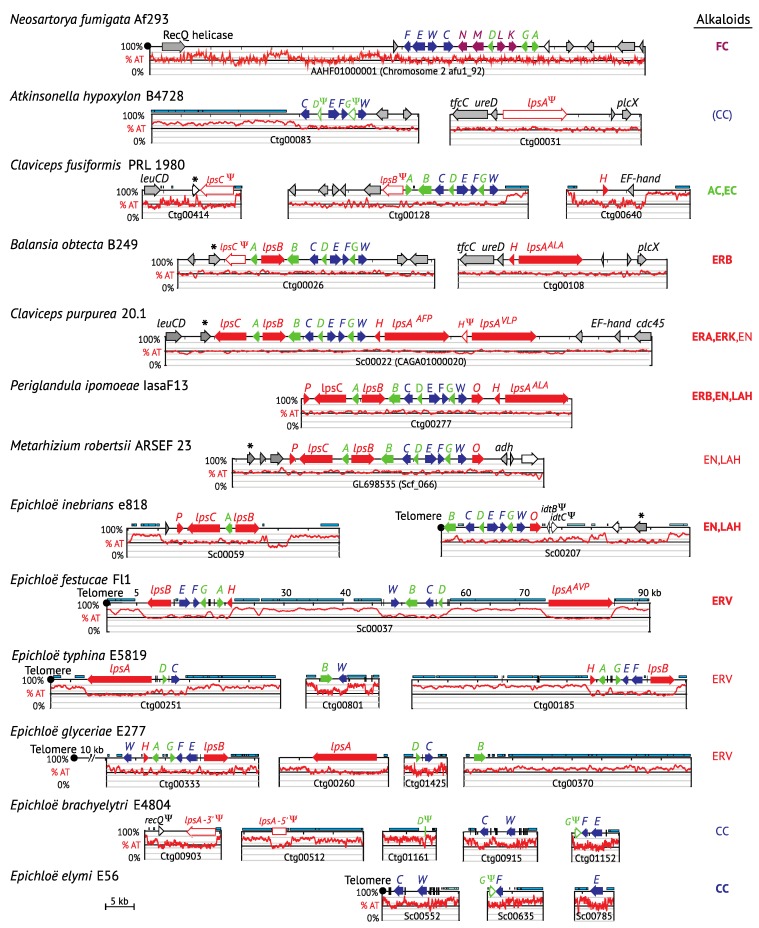
Relative adenine and thymine (%AT) DNA content of ergot alkaloid synthesis *(EAS)* loci. Gene name abbreviations are as follow: all *eas* genes = last letter, *cloA* = *B* and *dmaW* = *W*. Gene names are colored to represent the stage of the pathway for the encoded product (see Figure 1). Pseudogenes are represented by Ψ and white-filled arrows. The major pathway end product of each strain is indicated on the right in bold face (product produced) or regular type (product predicted but not yet tested), or in parentheses (product predicted but undetected). Arrows marked with * represent orthologues of *C. purpurea* AET79176 (GenBank). Cyan bars indicate repeats, and vertical black bars indicate miniature inverted-repeat transposable elements (MITEs). Where present, telomeres are positioned at left.

All known ergot alkaloid biosynthesis genes are present in the genomes of ascomycetous fungi grouped either in a single ergot alkaloid synthesis (*EAS*) cluster or divided into two *EAS* clusters (Figure 2). The particular forms and overall profile of ergot alkaloids produced by a fungus are determined by the presence or absence of pathway genes and, for several *EAS* genes, the particular enzyme isoforms they encode. The basic functions have been determined for all known *EAS* genes in the Clavicipitaceae, and most in the Trichocomaceae (reviewed in [20]), so that detecting gene presence or absence provides considerable power to predict alkaloid profiles, but there is also important variation in substrate and product specificities of some *EAS* gene*-*encoded enzymes, which we are not yet able to infer from the gene sequences [17].

### 2.1. Genes Encoding the Early Pathway Steps

The four conserved early pathway steps for all natural ergot alkaloids produce chanoclavine I (CC) (recently reviewed [20]), and are catalyzed by enzymes encoded in the four genes: *dmaW*, encoding dimethylallyltryptophan synthase, *easF*, encoding dimethylallyltryptophan *N*-methyltransferase, *easC*, encoding a catalase, and *easE*, encoding chanoclavine-I synthase. Some strains, such as *E. elymi* E56, produce CC as the pathway end-product because they contain functional copies only of these four genes in an *EAS* cluster, which we designate as *EAS*^CC^ (Figure 2; Table 1). Based on similar complements in their genomes, we would predict that *E. brachyelytri* E4804 and *Atkinsonella hypoxylon* B4728 are also CC producers, though CC has not been detected in plants infected with B4728. Strains capable of making complex ergot alkaloids also can generate substantial levels of CC and other intermediates and spur products as a result of inherent pathway inefficiency, a property suggested to have selective advantage [21,22].

**Table 1:** Ergot alkaloid synthesis *(EAS)* gene names and encoded functions.

Gene name ^a^	Enzyme	*EAS^CC^*	*EAS^EC^*	*EAS^ERP^*	*EAS^LAH^*	*EAS^EN/ERP^*	*EAS^LAH/ERP^*	*EAS^FC^*
*dmaW*	Dimethylallyltryptophan synthase	+	+	+	+	+	+	+
*easF*	Dimethylallyltryptophan*N*-methylase	+	+	+	+	+	+	+
*easC*	Catalase	+	+	+	+	+	+	+
*easE*	Chanoclavine-I synthase	+	+	+	+	+	+	+
*easD*	Chanoclavine-I dehydrogenase		+	+	+	+	+	+
*easA* ^b^	Chanoclavine-1 aldehyde oxidoreductase		+ iso	+ iso	+ iso	+ iso	+ iso	+ red
*easG*	agroclavine, festuclavine or pyroclavine dehydrogenase		+	+	+	+	+	+
*cloA* ^b^	agroclavine, festuclavine, or elymoclavine monooxygenase		+	+	+	+	+	
*lpsB*	lysergyl peptide synthetase subunit 2			+	+	+	+	
*lpsA* ^b^	lysergyl peptide synthetase subunit 1			+		+	+	
*easH*	ergopeptide lactam hydroxylase			+		+	+	
*lpsC*	lysergyl peptide synthetase and reductase subunit				+	+	+	
*easO* ^c^	putative ergonovine oxygenase				+		+	
*easP* ^c^	putative LAH synthase				+		+	
*easM* ^c^	possible festuclavine 9-monooxygenase							+
*easK* ^c^	possible festuclavine 9-monooxygenase							+
*easN*	fumigaclavine acetylase							+
*easL*	fumigaclavine reverse prenyltransferase							+
Fungal species	Examples of ergot alkaloid-producing fungi	*Epichloë elymi*	*Claviceps fusiformis*	*Epichloë festucae*,*Balansia obtecta*	*Epichloë inebrians*	*Claviceps purpurea*	*Periglandula ipomoeae*	*Neosartorya fumigata*

^a^ Pathway steps are color-coded based on the positions within the pathway as shown in Figure 1; ^b^ Specificity of encoded gene can vary. EasA functions as either an isomerase “+ iso” or reductase “+ red”; ^c^ Actual role not confirmed.

### 2.2. Diversification of the EAS Pathways

Beyond the production of CC, multiple biosynthetic pathways begin to branch and diverge (Figure 1), and the chemotypic variation between and even within species is reflected in the gene content of each *EAS* locus known or predicted to direct biosynthesis of such pathway end-products as elymoclavine (EC), lysergic acid α-hydroxyethylamide (LAH), ergonovine (EN), and ergopeptines such as ergovaline (ERV), ergotamine (ERA), ergocryptine (ERK) or ergobalansine (ERB). Where clarification is needed, we will designate the various *EAS* clusters with superscripts reflecting end products, as *EAS^EC^*, *EAS^LAH^* or *EAS^EN^*, as well as *EAS^ERP^* for ergopeptine producers, *EAS^EN/ERP^* for producers of EN and ergopeptines, and *EAS^LAH/ERP^* for producers of LAH and ergopeptines. Compared to *EAS^EN^*, *EAS^LAH^* has two additional genes, *easO* and *easP*, suggesting that EN may be the LAH precursor. Fungi with *EAS^LAH^* clusters also tend to accumulate substantial levels of ergine, probably by spontaneous hydrolysis of LAH [23]. The *EAS^EN/ERP^* cluster in the most infamous ergot fungus, *Claviceps purpurea*, and the *EAS^LAH/ERP^* cluster in the morning-glory symbiont, *Periglandula ipomoeae*, are the only ones identified to date that determine synthesis of three different ergot alkaloid subclasses [24].

#### 2.2.1. Completion of the Tetracyclic Ergolene Common Core

The EAS pathway diversifies at multiple steps depending, not only on presence or absence of genes, but also on the substrate- and product-specificities of several of the encoded enzymes [17,20] (Figure 1). Once CC is oxidized by the action of the EasD enzyme to form chanoclavine aldehyde, EasA then catalyzes a reduction step that allows rotation around the C8-C9 bond so that an iminium ion (*i.e.*, Schiff base) can form as the first step in the synthesis of the D-ring of the ergolene core common to most ergot alkaloids. Surprisingly, this is one of the steps at which different pathways can diverge to give either ergot alkaloids or dihydroergot alkaloids [17,25]. EasA proteins that follow the reduction step with reoxidation are effectively isomerases, typically found in *Claviceps purpurea* and many species of *Balansia*, *Epichloë* and *Periglandula*. In contrast, EasA isoforms that only reduce the C8-C9 bond direct the pathway toward dihydroergot alkaloids, such as those found in *Claviceps africana* and *Claviceps gigantea* as well as in the fumigaclavine producer, *Neosartorya fumigata*.

The ergolene D-ring is completed by a reduction catalyzed by EasG, to yield agroclavine (for ergot alkaloids) or festuclavine (for dihydroergot alkaloids) [20] (Figure 1). After the D-ring is closed, the C8-linked methyl group can be oxidized by the action of a cytochrome P450 monooxygenase. This enzyme, designated CloA, apparently represents another point in the pathway where variation in an enzyme can affect the alkaloid profile [17]. It appears likely that different isoforms of CloA determine the level of oxidation, such that CloA of *C. fusiformis* catalyzes the 2-electron oxidation of agroclavine (AC) to EC, whereas CloA of *C. purpurea* and many other Clavicipitaceae catalyzes a 6-electron oxidation of agroclavine to paspalic acid or lysergic acid (LA). Whether LA is generated spontaneously or enzymatically from paspalic acid remains unclear.

#### 2.2.2. Formation of Lysergic Acid, Lysergic Acid Amides and Complex Ergopeptines

Despite its fame as a starting material for laboratory synthesis of LSD, LA does not generally occur in appreciable concentrations in natural systems [22]. This is because fungi that make LA are usually capable of converting it to any of a multitude of lysergic acid amides, ranging from the simplest (ergine = lysergic acid amide) to complex ergopeptines in which LA has an amide linkage with a tricyclic moiety derived from three additional amino acids (Figure 1) (reviewed in [17]). This divergence point involves a remarkable system that centers on the enzyme subunit LpsB (=LPS2) plus one or both of its partner subunits LpsA (=LPS1) or LpsC (=LPS3) depending on whether functional *lpsA* or *lpsC* genes are present [26]. Each of the Lps subunits contains modules that contribute specific catalytic activities and, in combination with other Lps subunits, comprise multi-enzyme complexes called nonribosomal peptide synthetases (NRPSs). Each module AMPylates and thio-esterifies an amino acid, and then condenses it with the similarly processed amino acid on the adjacent module. LpsB specifies LA, whereas LpsC specifies l-alanine (Ala). LpsC also has a C-terminal “R*” domain (cd05235: SDR_e1) that catalyzes reductive release of the LA-Ala conjugate as EN. Alternatively, if LpsB partners with LpsA they form the enzyme complex required for formation of ergopeptide lactams, which are then oxidatively cyclized by the action of EasH [27] to form ergopeptines or their 8(*R*) isomers, the ergopeptinines. The particular composition of each ergopeptine is determined by specificity of each of the three modules in LpsA for its cognate amino acid [28], so far giving 21 different combinations (Table 2) [19,29,30]. For example, *Epichloë* strains that produce ergovaline (ERV) have LpsA^AVP^ (where the superscripts are single-letter codes for the amino acids specified, in order, by the first, second and third modules of LpsA). In contrast, *C. purpurea* strain 20.1 has genes for the LpsA isoforms LpsA^AFP^ and LpsA^VLP^, which determine production of ergotamine (ERA) and ergocryptine (ERK), respectively. There are 19 ergopeptines known, most with corresponding ergopeptinines, which are their 8(*S*) stereoisomers. Two additional ergopeptinines have no known 8(*R*) isomers (reviewed in [30]). Additionally, *Claviceps africana* produces dihydroergosine, which is similar to ergosine except that it has a saturated D-ring, presumably because it is derived from festuclavine rather than agroclavine (reviewed in [17]).

#### 2.2.3. Fumigaclavine Production by the Trichocomaceae

The Trichocomaceae produce various clavines derived from festuclavine (reviewed in [17]) (Figure 1). They lack agroclavine or its derivatives because they have a reducing rather than isomerizing EasA isoform. They may also produce pyroclavine, the 8(*S*) stereoisomer of festuclavine, due to functional differences in EasG [31]. Further modifications are catalyzed by enzymes encoded in the *EAS* cluster of *N. fumigata*, for which orthologs are not found in other fungi, giving rise to the fumigaclavines. The 9-hydroxylation is probably catalyzed by either EasM or EasK, both predicted to be cytochrome P450. Then *O*-acetylation is catalyzed by EasN, and the “reverse” prenylation step is catalyzed by EasL.

**Table 2:** Lysergic acid-linked substituents of natural ergopeptines ^a^.

Ergopeptine	AA1	R^1^	AA2	R^2^	AA3	R^3^
Ergotamine (ERA)	Ala	Me	Phe	CH_2_Ph	Pro	prolyl (CH_2_)_3_
Ergovaline (ERV)	Ala	Me	Val	*i*-Pr	Pro	prolyl (CH_2_)_3_
Ergosine	Ala	Me	Leu	*i*-Bu	Pro	prolyl (CH_2_)_3_
Dihydroergosine ^b^	Ala	Me	Leu	*i*-Bu	Pro	prolyl (CH_2_)_3_
β-Ergosine	Ala	Me	Ile	*sec*-Bu	Pro	prolyl (CH_2_)_3_
Ergosedmine	Ile	*sec*-Bu	Leu	*i*-Bu	Pro	Prolyl (CH_2_)_3_
Ergobine	Ala	Me	ABA	Et	Pro	prolyl (CH_2_)_3_
Ergocristine	Val	*i*-Pr	Phe	CH_2_Ph	Pro	prolyl (CH_2_)_3_
Ergocornine	Val	*i*-Pr	Val	*i*-Pr	Pro	prolyl (CH_2_)_3_
Ergocryptine ^c,d^ (ERK)	Val	*i*-Pr	Leu	*i*-Bu	Pro	prolyl (CH_2_)_3_
β-Ergocryptine ^d^	Val	*i*-Pr	Ile	*sec*-Bu	Pro	prolyl (CH_2_)_3_
γ-Ergocryptinine ^c,e^	Val	*i*-Pr	norLeu	*n*-Bu	Pro	prolyl (CH_2_)_3_
Ergobutyrine	Val	*i*-Pr	ABA	Et	Pro	prolyl (CH_2_)_3_
Ergoladinine ^e^	Val	*i*-Pr	Met	EtSCH_3_	Pro	prolyl (CH_2_)_3_
Ergogaline	Val	*i*-Pr	homoIle	2-Me- *n*-Bu	Pro	prolyl (CH_2_)_3_
Ergostine	ABA	Et	Phe	CH_2_Ph	Pro	prolyl (CH_2_)_3_
Ergonine	ABA	Et	Val	*i*-Pr	Pro	prolyl (CH_2_)_3_
Ergoptine ^c^	ABA	Et	Leu	*i*-Bu	Pro	prolyl (CH_2_)_3_
β-ergoptine	ABA	Et	Ile	*sec*-Bu	Pro	prolyl (CH_2_)_3_
Ergobutine	ABA	Et	ABA	Et	Pro	prolyl (CH_2_)_3_
Ergobalansine (ERB)	Ala	Me	Leu	*i*-Bu	Ala	Me
Unnamed, from *Dicyma* sp.	Ala	Me	Leu	*i*-Bu	Phe	CH_2_Ph

^a^ Abbreviations: AA= amino acid position; ABA = 2-aminobutyric acid, norLeu = l-norleucine; homoIle = l-homoisoleucine. Other l-amino acids and R-groups are abbreviated as standard; ^b^ Dihydroergosine has a saturated D-ring, whereas others listed here have a 9-10 double bond; ^c^ Synonyms: ergosine = α-ergosine, ergocryptine = α-ergocryptine, ergoptine = α-ergoptine; ^d^ Synonyms: α-, β-, or γ-ergocryptine = α-, β-, or γ-ergokryptine, respectively; ^e^ Only the 8(*R*) (=isolysergyl) isomers—namely, ergoladinine and γ-ergocryptinine—have been reported to date.

### 2.3. Contents of the EAS Loci

The EAS pathway specificity mentioned above is reflected in the structural content of *EAS* loci across the Clavicipitaceae, which varies based on presence or absence of genes that correspond to ergot alkaloid biosynthetic capability within a given strain. The most *EAS* genes (14) are present in *P. ipomoeae*, which produces ergobalansine (ERB), EN and LAH, whereas only four *EAS* genes are common to all strains that are capable or predicted to produce CC [24]. Interestingly, those four early-pathway genes are grouped together in the *N. fumigata* cluster, whereas the mid-pathway genes for festuclavine biosynthesis are interspersed with late-pathway genes for fumigaclavine [32,33] (Figure 2). The gene arrangements differ between *N. fumigata* and the Clavicipitaceae, and also differ considerably between *Epichloë* spp. and other Clavicipitaceae. In *At. hypoxylon*, *Balansia obtecta*, the *Claviceps* spp., *Epichloë inebrians* (formerly *Epichloë gansuensis* var. *inebrians*; [34]) and *P. ipomoeae*,early and mid-pathway genes (except for *easA*) are interspersed, whereas the late genes (*lps* genes, *easH*, *easO* and *easP*) are at the *EAS*-cluster periphery, separated from the main *EAS* cluster, fatally mutated or missing. The *easA* gene is the exception among mid-pathway genes, being located between late-pathway genes *lpsB* and *lpsC* in these species. In the other *Epichloë* species, there is similar variation in gene content and functionality, but greater variation in gene arrangement even for early- and late-pathway genes. Clearly, strains that produce only CC are derived by losses of mid-and late-pathway genes, because remnants and pseudogenes often remain in their genomes.

Putative *EAS* genes can be identified in fungal genome sequences using bioinformatics pipelines developed to identify core secondary metabolite genes encoding NRPSs, polyketide synthases (PKS), prenyltransferases (*dmaW* homologues) or terpene cyclases and mixed or hybrid versions [35,36,37]. The *EAS* gene clusters such as *EAS^CC^* can sometimes be identified within the prenyltransferase or terpene cyclase group, whereas a more complex *EAS* cluster, such as *EAS^ERP^*, will fall into a hybrid or mixed category since they contain sequences for both prenyltransferases and NRPSs [38]. In genomes of several *Trichophyton* and *Arthroderma* species (Arthrodermataceae), apparent orthologues of *dmaW*, *easF*, *easC*, *easE* and *easD* are identifiable in a cluster [18]. Genes flanking this cluster match signatures of various biosynthetic functions, so it may well be that Arthrodermataceae produce one or more alkaloid subclasses yet to be identified. The *EAS* gene complements and structural arrangements identified in *Metarhizium robertsii* [39] are very similar to *E. inebrians* and, as such, we would predict that *M. robertsii* strain ARSEF 23 could be capable of producing EN and LAH, assuming there is similar specificity of LpsB with LA and LpsC with alanine.

## 3. Phylogenetic Relationships of *EAS* Genes

### 3.1. Comparison of *EAS* Gene Phylogenies

**Figure 3:**
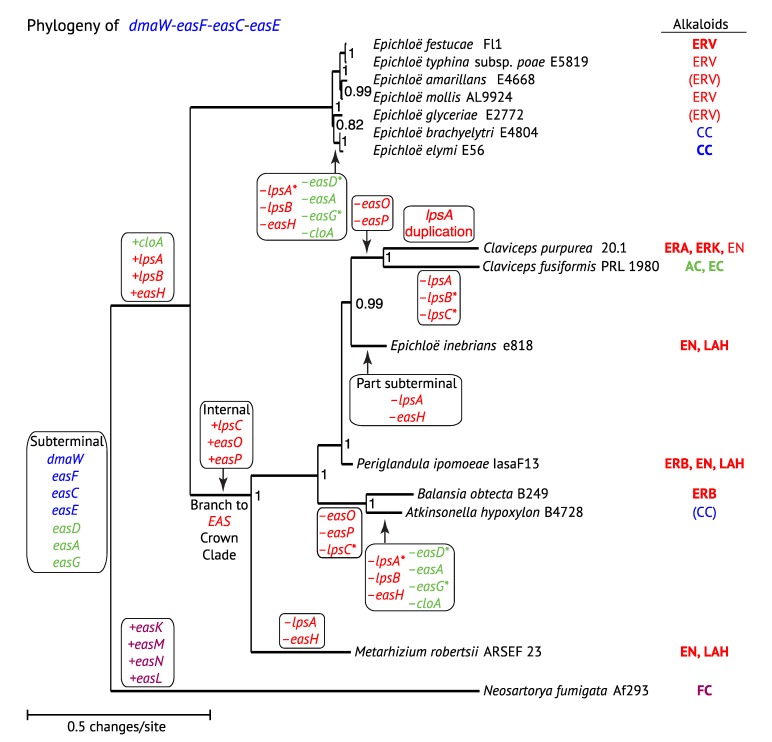
Phylogeny of concatenated *dmaW*-*easF*-*easC*-*easE* genes. The phylogenetic tree is based on a nucleotide alignment of coding sequences of the core genes for the first four steps in ergot alkaloid biosynthesis available from sequenced genomes. Sequences were aligned with MUSCLE [41], and trees were inferred by maximum likelihood with PhyML implemented by Phylogeny.fr [40]. Node support was determined by the approximate likelihood ratio test [42]. Gene gains and loses are indicated by + and –, respectively, and asterisks (*) indicate that remnants or pseudogenes can be found in one or more members of the clade. Genes are color-coded based on position of the encoded step within the pathway. The major pathway end product of each strain is indicated on the right in bold face (product produced) or regular type (product predicted but not yet tested), or in parentheses (product predicted but undetected).

**Figure 4:**
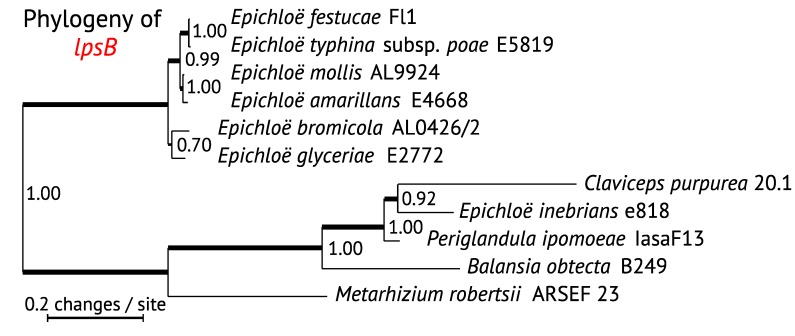
Phylogeny of lysergyl peptide synthetase subunit 2 (*lpsB)*. The phylogenetic tree was inferred by maximum likelihood on a nucleotide alignment of coding sequences. Methods are as in Figure 3. The left edge is placed to correspond to the root inferred in Figure 3 with *Neosartorya fumigata EAS* genes as the outgroup; *N. fumigata* lacks *lpsB*.

**Figure 5:**
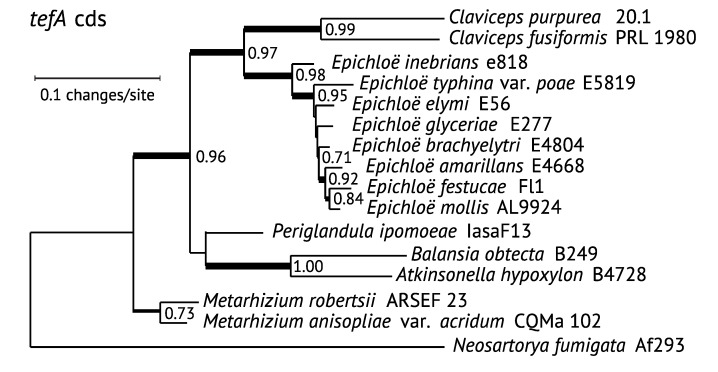
Phylogeny of *tefA*, encoding translation elongation factor 1-α. The phylogenetic tree inferred by maximum likelihood on a nucleotide alignment of coding sequences. Methods are as in Figure 3.

We have inferred phylogenetic trees for *EAS* genes of known and suspected ergot alkaloid-producing Clavicipitaceae, plus *N. fumigata*, which we presumed to be an outgroup in keeping with housekeeping gene relationships. Phylogenies of the core genes for the first four steps in ergot alkaloid biosynthesis—namely, *dmaW*, *easF*, *easC* and *easE*—were congruent, so a concatenated data set (*WFCE*) was constructed, from which a maximum likelihood (ML) tree was inferred with PhyML at phylogeny.fr [40] (Figure 3). The *lpsB* tree (Figure 4) was also congruent with the corresponding *WFCE* subtree (*i.e.*, the tree pruned of taxa lacking *lpsB*). The *WFCE* and *lpsB* phylogenies were significantly supported at all nodes. The *WFCE* phylogeny grouped sequences from seven out of eight *Epichloë* species in a clade that was basal in the Clavicipitaceae, whereas the sequences from the eighth representative, *E. inebrians*, appeared as the sister to the *Claviceps* clade. Comparing this phylogeny to that of the housekeeping gene, *tefA* (Figure 5), the only significant disparity was the basal placement of the main clade of *Epichloë EAS* genes, which contrasted with the *tefA* phylogeny that placed all of the *Epichloë* species together in a clade with a sister relationship to the *Claviceps* clade. There was also a possible disparity in placement of *P. ipomoeae*, but this cannot be considered significant because the relevant branch in the *tefA* tree lacked statistical support. Therefore, with the glaring exception of the main *Epichloë EAS* clade, evolution of *EAS* genes in the Clavicipitaceae appears to have been by direct decent without duplication (paralogy) or horizontal gene transfer.

The well-supported position of *EAS* genes from most *Epichloë* species, being basal among the Clavicipitaceae (Figure 3 and Figure 4) indicates a deviation from strict orthology because it differs dramatically from housekeeping gene phylogenies (Figure 5). Possible causes of this deviation are trans-species polymorphism, paralogy or horizontal gene transfer. In a BLASTp search of *dmaW* against available sequences at GenBank, no homologues were closely related to this clade except those of other *Epichloë* species, so there was no obvious source for a horizontal gene transfer event. Comparing the genomic context, sequences nearest the *EAS* clusters differed between *E. festucae* and *E. inebrians* (assemblies of other *Epichloë* species did not link *EAS* clusters with other genes), so trans-species polymorphism also was unsupported. This leaves, as our favored possibility, that the *E. inebrians* and other *Epichloë EAS* clusters were derived from paralogous copies that arose from duplication of the *EAS* cluster in an ancestor to most or all of the Clavicipitaceae. In this regard, the *tefA* phylogeny (Figure 5) placed *E. inebrians* basal in genus *Epichloë*, supporting the possibility that the cluster common to most *Epichloë* species was lost on that basal branch to *E. inebrians*. However, a puzzle remains in that no genus other than *Epichloë* showed indications of paralogous *EAS* clusters. This may be just a matter of limited sampling, and we predict that a wider and deeper survey of the Clavicipitaceae will reveal paralogs related to the basal group of *Epichloë EAS* sequences.

Paralogous *dmaW* genes are in fact evident in some of the Clavicipitaceae. Specifically, *C. purpurea* 20.1 has two *dmaW* copies flanking a paralogous *easF* and located 94 kb and 98 kb from the *EAS*-cluster *dmaW*. *Epichloë mollis* also has a paralogous *dmaW*, which appears to be a pseudogene. However, these paralogues are due to relatively recent duplications and group with the respective *Claviceps* and *Epichloë dmaW* genes in phylogenetic analysis [43].

### 3.2. Mapping EAS Gene Gains and Losses

Mapping genomic alternations onto the *WFCE* phylogeny (Figure 3) revealed repeated instances in which multiple *EAS* genes have been lost. The most extensive losses were eight genes in two separate instances resulting in gene sets for CC production: The branch to *E. elymi* and *E. brachyelytri*, and the branch to *At. hypoxylon*. The identification of remnant or pseudogene copies of other *EAS* genes supported the scenario of extensive gene loss, as inferred from the phylogeny. Losses of multiple *EAS* genes on numerous lineages have given rise to at least four distinct chemotypes in addition to the variations in ergopeptines. These gene losses add to the potential for ergot alkaloid diversification, together with neofunctionalization of *lpsA* (Table 2), and with *easA* variations to give agroclavine or festuclavine as precursors of ergot alkaloids and dihydroergot alkaloids, respectively, and *easG* variations to yield 8(*S*)-dihydroergot alkaloids (Figure 1) [17].

Interestingly, in almost all cases of *EAS* gene loss (Figure 3), all genes were lost or inactivated for a branch of the pathway, leaving only those *EAS* genes required for biosynthesis of the observed metabolites. In two instances, this process has given CC as the end product (or presumed end product), in one instance it has given EN rather than LAH, and in three instances it has eliminated the ergopeptine pathway. The only exception to this pattern is in *C. fusiformis*, which has apparently retained *easH* despite losing all other genes for late pathway steps both to ergopeptines and to EN and LAH. However, whether *easH* is transcribed or gives an active (but presumably useless) product in *C. fusiformis* is unknown. Among many natural strains, gene losses are similarly evident in indole-diterpene (*IDT*) and loline alkaloid (*LOL*) gene clusters with loss or inactivation of all other genes for downstream steps of the affected pathway branch [24,44,45]. Such common patterns suggest that there is selection against expression of enzymes in strains that lack their normal substrate. We speculate that expression of such enzymes may be directly harmful because the enzymes may catalyze reactions with substrates other than the missing natural substrate, thereby generating toxic products.

### 3.3. Positional Changes of Clusters with Respect to Telomeres

Also mapped to the *WFCE* phylogeny (Figure 3) are three changes in the *EAS* cluster positions relative to telomeres. Considering that the *N. fumigata* and basal *Epichloë* *EAS* genes are near telomeres (“subterminal”), it is parsimonious to propose this as the ancestral state. If so, internalization would have occurred on the branch that separates most of the Clavicipitaceae from the basal *Epichloë EAS* genes. An interesting translocation event is evident on the branch to *E.* *inebrians*, whereby the *EAS* cluster was divided and at least one of the two resulting portions returned to a subterminal position. Interestingly, the *cloA* gene (designated *B* on the map in Figure 2) is situated such that its stop codon is a single base away from the telomere repeat array, implying that the telomere serves as a transcription terminator for that gene. The translocation event on the *E. inebrians* branch nicely illustrates the dynamics of subterminal regions of the chromosomes. Recalling that the *IDT* cluster—directing biosynthesis of the indole-diterpene, paxilline—is subterminal in the closely related *E. gansuensis* strain E7080 [24], it is particularly interesting to find two remnant *IDT* genes flanking the centromeric side of the subterminal *EAS* cluster in *E. inebrians*. It appears likely that the *EAS* cluster displaced most of the *IDT* cluster as it moved into the subterminal position. This supports an important role of subterminal regions in arrangements and rearrangements of secondary metabolism gene clusters. With that in mind, we would predict that most *EAS* cluster rearrangements occur in association with subterminal clusters, whereas internal *EAS* clusters are more stable. Evidence pertinent to that prediction is discussed later, in Section 5.

### 3.4. Evolution of LpsC

**Figure 6:**
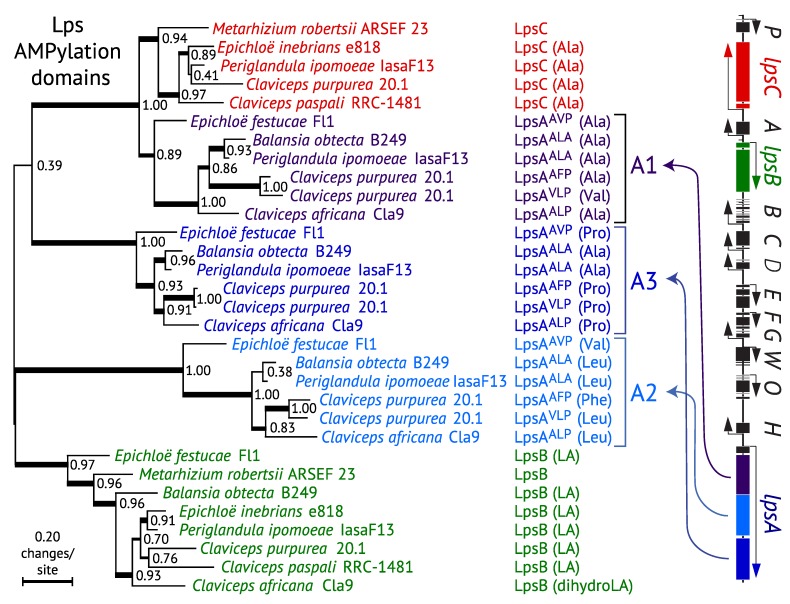
Phylogeny of the Lps subunit AMPylation domains. The phylogenetic tree was inferred by maximum likelihood on a nucleotide alignment of coding sequences. Methods are as in Figure 3. The specified substrates are given in parentheses as LA = lysergic acid, dihyroLA = dihydrolysergic acid, and standard abbreviations for common l-amino acids. The LpsA superscripts indicate single-letter codes for the amino acids specified by AMPylation domains of module 1, 2 and 3 (A1, A2 and A3), respectively. Functionality and specificity of *M. robertsii* LpsB and LpsC are unknown. The *P. ipomoeae* *EAS* cluster is shown at right with Lps genes and modules color-coded.

Assuming that the common ancestor of Clavicipitaceae and Trichocomaceae possessed the seven genes for early and intermediate biosynthetic steps, a parsimonious scenario would identify three branches for gene gains: one to *N. fumigata*, one to all Clavicipitaceae, and the third to Clavicipitaceae after splitting from the basal *Epichloë EAS* clade (Figure 3). In the context of our proposed paralogy, the last of these would have involved acquisition of the new genes, *lpsC*, *easO* and *easP*, on one of the branches after that split in the Clavicipitaceae. Interestingly, LpsC serves as an alternative to the LpsA subunit in interacting with LpsB as a component of a lysergyl peptide synthetase complex (Figure 1). A phylogenetic analysis of Lps subunit A domains (Figure 6) indicates that *lpsC* may be derived from a copy of the first module of *lpsA* fused to an R* (reductase) coding sequence. Thus, biosynthesis of EN and LAH seems to have evolved later than biosynthesis of the much more complex ergopeptines.

### 3.5. Evolution of Module Specificity in LpsA

The evolution of module specificity in LpsA subunits can also be mapped onto the *WFCE* (Figure 3) and *lpsA* A-domain (Figure 6) phylogenies. However, a crucial difference between *WFCE* and *lpsA* phylogenies is that *lpsA* A-domains consistently group *P. ipomoeae* and *B. obtecta* in a terminal clade, whereas *WFCE* groups *P. ipomoeae* with *Claviceps* spp. and places *B. obtecta* in a relatively basal position. This may be the result of *lpsA* paralogy due to duplications and losses (a scenario reminiscent of the tandemly duplicated *lpsA* genes in *C. purpurea*), or may simply be due to lineage sorting effects in the evolution of these lineages (as suggested by the short and poorly supported branch in the *tefA* phylogeny). To discuss LpsA module evolution, we now use single-letter abbreviations for the three specified amino acids in order of the modules 1, 2 and 3. The ancestral state could have been ALP or AVP, with module A2 changing specificity either on the basal *Epichloë* branch or on the lineage leading to the *Claviceps*/*Periglandula*/*Balansia* clade, respectively. During evolution of the *Periglandula*/*Balansia* clade, specificity of module 3 switched from P to A. What is especially interesting is that, within *Claviceps*, there is an accelerated diversification of modules 1 and 2, such that the variant LpsA subunits specify the substrate combinations for at least 19 different ergopept(in)ines (Table 2).

## 4. Ergot Alkaloid Diversity within *Epichloë* Species

### 4.1. Distribution of EAS Genes across Epichloë Species

The distribution of the *EAS* locus is well understood within *Epichloë* species due to the availability of genomic and genotyping data (www.endophyte.uky.edu). Genome sequences (including draft genomes) for 54 isolates representing 10 described *Epichloë* species, two varieties, and five undescribed species have enabled comparisons of the *EAS* locus across a diverse collection of isolates with varying capabilities to produce ergot alkaloids [24,34,38,43,45,46]. Moreover, in recent studies, markers developed from the genome sequences have been used for PCR-based genotyping of endophyte-infected grass collections for the presence of genes encoding key alkaloid biosynthesis pathway steps [10,47,48,49,50,51,52]. Compared to genome sequencing, PCR-based genotyping is less reliable in determining if a gene is functional, but the presence of full-length genes typically associated with *EAS* clusters for the metabolites CC, ERV or EN usually corresponds well to the production of the corresponding metabolite. The predictive power of this method is helped by the tendency for complete losses or large deletions in the nonfunctional alkaloid biosynthesis genes, and for the downstream genes to be partly or completely deleted as well [24].

The presence of *EAS* genes within *Epichloë* species has been observed in strains of at least 19 (9 nonhybrid and 10 hybrid species) of the 35 taxa tested (including varieties and undescribed species) (Table 3). The *EAS* locus has a discontinuous distribution within *Epichloë* species. For example, not all *E. festucae* isolates are capable of producing ergovaline, since some isolates (e.g., *E. festucae* E434) lack the *EAS* locus. However, in addition to their discontinuous distribution, the *EAS* loci can differ in structural content. Strains capable of producing CC (e.g., *E. elymi* E56) only contain functional genes encoding the first four *EAS* pathway steps (DmaW, EasF, EasC and EasE) (Figure 1; Table 1), whereas ergovaline producers (e.g., *E. festucae* Fl1) have more complex *EAS* loci (*EAS^ERP^*) with at least 11 functional genes present (Table 1).

Many of the chemotype differences identified within the *Epichloë* species can be attributed to the presence or absence of genes encoding the key pathway steps, but there are some anomalies. Isolates, such as *E. festucae* strains E2368 and E189, *E. amarillans* E4668 and *E. glyceriae* E2772 appear to have complete *EAS*^ERP^ clusters with no obvious deleterious mutations, yet ergot alkaloids have not been detected in host plants symbiotic with these isolates. Transcriptome (RNA-seq) analysis of E2368-infected meadow fescue (*Lolium pratense*) and tall fescue show that the *EAS* genes are not expressed in this strain, thus providing a clue to why the predicted alkaloid, ERV, is not produced [24].

Many endophytes can readily produce ergot alkaloids *in planta* but have a more limited and unreliable production in non-symbiotic culture conditions [53,54]. Histone methylation apparently helps repress expression of *EAS* and other alkaloid gene clusters when *Epichloë* species are grown in culture, since the *EAS* and *LTM* genes of *E. festucae* strain Fl1 were de-repressed under non-symbiotic culture conditions when two genes that encode histone H3 methylases were deleted [55].

**Table 3:** *EAS* gene distribution within and between *Epichloë* species.

*Epichloë* species ^a^	Host species	Detection method ^b^	*EAS* gene variations (strains observed) ^c^	Reference
*Epichloë amarillans*	*Agrostis hyemalis*	GT, DG, G	0* (4), (ERV) (1)	[24]
*E. aotearoae*	*Echinopogon ovatus*	G	0 (1)	[24,43]
*E. baconii*	*Agrostis tenuis*,*Calamagrostis villosa*	GT, G	0* (3)	[43]
*E. brachyelytri*	*Brachyelytrum erectum*	GT, G	0 (1), CC (3)	[24]
*E. bromicola*	*Bromus erectus*,*Bromus benekenii*,*Bromus tomentellus*,*Agropyron hispidus*	GT, DG, G	0* (5)	[43]
*E. cabralii* (H)	*Phleum alpinum Bromus laevipes*	G, GT	0 (1), (ERV) (2)	[50]
*E. canadensis* (H)	*Elymus canadensis*	GT, DG	**CC (1), ERV (1)**	[43,47]
*E. chisosa* (H)	*Achnatherum eminens*	DG	0 (1)	[43]
*E. coenophiala* (H)	*Lolium arundinaceum*	GT, DG	0* (11), **ERV (12)**, ERV (39)	[43,49,51]
*E. elymi*	*Elymus virginicus*	GT, G	0 (1), **CC (1)**	[24]
*E. festucae*	*Festuca trachyphylla*,*Festuca rubra* subsp.*rubra*,*Lolium giganteum*	GT, G	0 (1), **ERV (1)**, (ERV) (2)	[24]
*E. festucae* var*. lolii*	*Lolium perenne*	GT, G	**ERV (2)**, (ERV) **(1)**	[56,57]
*E. festucae* var*. lolii x E. typhina* (H)	*Lolium perenne*	DG	**ERV (1)**	[43,58]
*E. funkii* (H)	*Achnatherum robustum*	GT, DG	**CC (1)**	[43]
*E. gansuensis*	*Achnatherum inebrians*	G	0 (1)	[24]
*E. inebrians*	*Achnatherum inebrians*	G	**EN, LAH (1)**	[24]
*E. glyceriae*	*Glyceria striata*	GT	(ERV) (2)	[24]
*E. mollis*	*Holcus mollis*	G	ERV (1)	[43]
*E. occultans* (H)	*Lolium* sp. (2x)	GT	0 (3)	[43]
*E. schardlii* (H)	*Cinna arundinacea*	GT	0 (1)	[59]
*E. siegelii* (H)	*Lolium pratense*	DG	0 (1)	[43]
*E. sylvatica*	*Brachypodium sylvaticum*	GT	0 (2)	[34]
*E. typhina*	*Lolium perenne*,*Dactylis glomerata*	G, GT	0 (3)	[24,43]
*E. typhina* ssp*. clarkii*	*Holcus lanatus*	GT	ERV (1)	unpublished
*E. typhina* ssp*. poae*	*Poa nemoralis*,*Bromus laevipes*	GT, G	0 (3), ERV (1)	[24,50]
*E. uncinata* (H)	*Lolium pratense*	DG	0 (1)	[43]
*E.* sp. AroTG-2(H)	*Achnatherum robustum*	GT	**EN (1)**	[10]
*E.* sp. BlaTG-3(H)	*Bromus laevipes*	GT	0* (1), **CC (2)**	[50]
*E.* sp. FaTG-2(H)	*Lolium* sp. (6x)	GT, DG	**ERV (10)**, ERV (33)	[43,49,51,60]
*E.* sp. FaTG-3(H)	*Lolium* sp. (6x), (8X)	GT, DG	0 (11)	[43,51,60]
*E.* sp. FaTG-4(H)	*Lolium* sp. (10x)	GT, DG	**ERV (1)**, ERV (11)	[43,51]
*E.* sp. FcaTG-1(H)	*Festuca campestris*	GT	0 (3)	unpublished
*E.* sp. FveTG-1(H)	*Festuca versuta*	GT	0 (2)	unpublished
*E.* sp. PalTG-1(H)	*Poa alsodes*	GT	0* (1)	unpublished
*E.* sp. PauTG-1(H)	*Poa autumnalis*	GT	0 (1)	unpublished

^a^ Endophytes that are known hybrids = (H); ^b^ Detection methods for the EAS genes DG = draft genome, G = genome, GT = PCR-based genotyping; ^c^ Number of independent strains evaluated. Alkaloids are abbreviated CC = chanoclavine I, ERV = ergovaline, EN = ergonovine, LAH = lysergic acid α-hydroxyethylamide, and are in bold face (product produced) or regular type (product predicted but not yet tested), or in parentheses (product predicted but undetected). 0 = No *EAS* genes identified, 0* = contains only remnants of *EAS* clusters; 0 and 0* are unable to produce ergot alkaloids.

### 4.2. Pseudogenes and Gene Remnants within the EAS Locus

**Figure 7:**
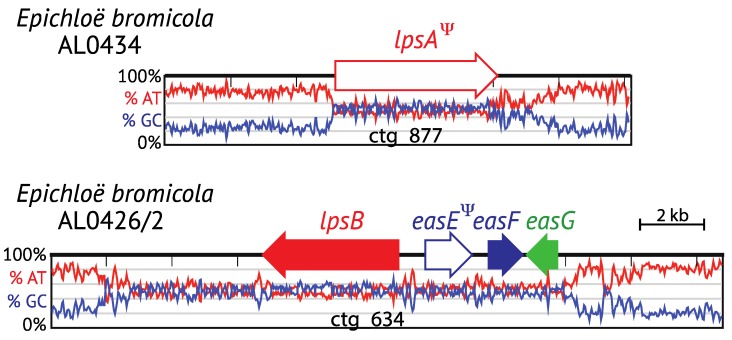
Remaining *EAS* genes and pseudogenes after independent losses in two *E. bromicola* isolates, AL0434 and AL0426/2. The AT-GC DNA contents are shown under the maps. Pseudogenes are represented by Ψ.

Pseudogenes and gene remnants have been identified within or adjacent to many of the described *EAS* clusters within the Clavicipitaceae. Most *Epichloë* species that are unable to produce ergot alkaloids lack functional *EAS* genes or only retain remnants of some *EAS* genes. For example, a remnant *lpsA* sequence is present in the genome sequences of *E. amarillans* strain E57, *E. bromicola* strain AL0434, and *Epichloë coenophiala* strains e4509, AR542 and AR584. Typically these *lpsA* remnants have multiple frameshifts, stop codons or both, and are flanked or even disrupted by AT-rich repeat sequences; or, in the case of E57, the gene is truncated at the telomere [24]. In comparison to *E. bromicola* strain AL0434, which has only an *lpsA* remnant, strain AL0426/2 has retained a greater number of *EAS* genes (Figure 7). The *E. bromicola* AL0426/2 genome assembly (www.endophyte.uky.edu) contains *lpsB*, *easE*, *easF* and *easG* (contig 634), but *easE* is a pseudogene in which part of the coding sequence is missing. Since *dmaW* and *easC* are also missing, the AL0426/2 isolate is not expected to produce ergot alkaloids. The identification of two independent *EAS* gene loss events in *E. bromicola* (strains AL0434 and AL0426/2) suggests that this species may have greater *EAS* diversity than is currently recognized, but *E. bromicola* strains capable of producing ergot alkaloids have not yet been identified [61].

### 4.3. Hybrids: EAS Gene Cluster Variations

**Figure 8:**
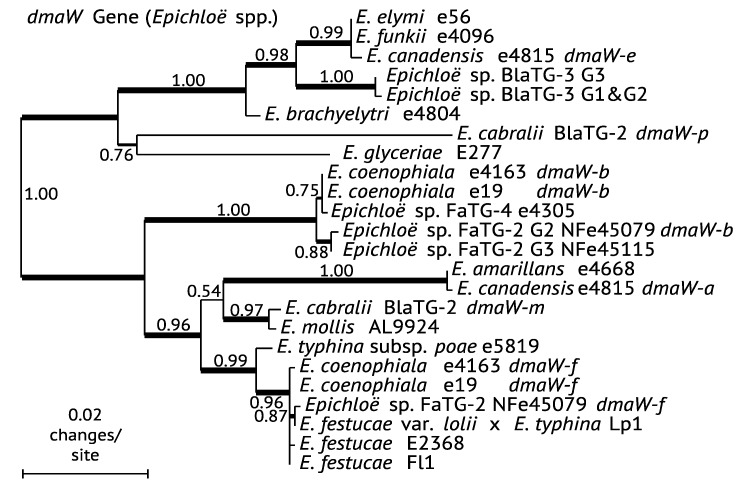
Phylogeny of *dmaW* genes of *Epichloë* strains. The phylogenetic tree was inferred by maximum likelihood on a nucleotide alignment of coding sequences. Methods are as in Figure 3. The *dmaW* alleles are distinguished in hybrids that possess more than one copy with a letter that refers to the ancestral progenitor (*a* = *E. amarillans*, *b* = *E. baconii*-related *Lolium* associated *Epichloë* subclade, *e* = *E. elymi*, *f* = *E. festucae*, *m* = *E. mollis*-related and *p = E. typhina* subsp. *poae.* The *dmaW* gene of *E. inebrians* has been omitted in this analysis because the gene and *EAS* locus is more similar to that of *P. ipomoeae* than to those of other *Epichloë* species (see Figure 3).

Compared to sexual strains or other haploids, the hybrid *Epichloë* species have a greater potential to contain *EAS* genes because each of the ancestors contributes genetic information. In addition, variations of the *EAS* locus within a hybrid can be due to copy number or differences within the inherited *EAS* cluster (*EAS^CC^* vs *EAS^ERP^* clusters). Some, but not all isolates from the hybrid species, *Epichloë canadensis*, *E. coenophiala*, and *E.* sp. FaTG-2, contain two *EAS* copies (Figure 8). The *E. canadensis* isolate e4815 contains two *EAS* clusters—*EAS^ERP^* for ERV and *EAS^CC^*—that are representative of the two contributing ancestors, *E. amarillans* and *E. elymi*, respectively. In contrast, *E. canadensis* isolate CWR34 contains a single *EAS^CC^* cluster, contributed by its *E. elymi* ancestor. Mating-type gene differences between *E. canadensis* isolates e4815 and CWR34 clearly indicate that they are the result of independent hybridizations, but it is unclear if CWR34 subsequently lost the *E. amarillans EAS* cluster, or if its particular ancestral strain of *E. amarillans* lacked *EAS* genes.

### 4.4. Gene Losses in Hybrids with Multiple Copies

**Figure 9:**
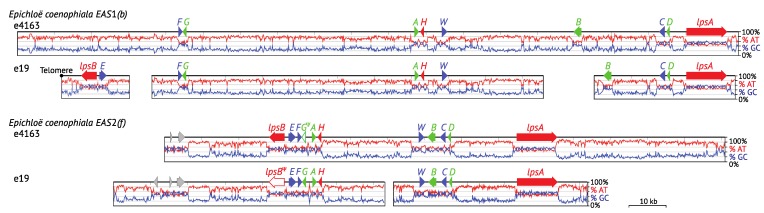
Structures of the *EAS* clusters from two *E. coenophiala* strains, e19 and e4163. The AT-GC contents are shown under the maps. Gene names are abbreviated as in Figure 2.

Some isolates of *E. coenophiala* are unable to produce ergot alkaloids because they only contain a remnant *EAS* locus (e.g., e4309, AR542 and AR584; Table 3) [51]. The *E. coenophiala* isolates known to produce ERV, such as isolates e19 and e4163, have two copies of the *EAS* clusters from two of the three contributing ancestors: *E. festucae* and the *Lolium*-associated *Epichloë* (LAE) subclade. Interestingly, the *EAS* clusters of both *E. coenophiala* isolates e19 and e4163 are structurally very similar with respect to AT content and repeat sequences, though they differ in which *EAS* genes are absent or inactive pseudogenes in each *EAS* cluster (Figure 9). The *lpsB*2 gene in e19 (from the *E. festucae* ancestor) is nonfunctional due to a frame shift within the coding region. The situation in e4163 is more complex in that neither *EAS* cluster is complete; *EAS*1 (from LAE) lacks *lpsB*1 and *easE*1, and *EAS*2 (from *E. festucae*) includes a nonfunctional *easG*2. Therefore, since e4163 is able to produce ERV, each *EAS* cluster must functionally complement the genetic deficiencies of the other cluster. *Epichloë* sp. FaTG-2 isolates NFe45115 and NFe45079 are both capable of producing ERV, but *EAS* copy numbers differ; NFe45115 has one copy, whereas NFe45079 has two copies [49]. Recent genome sequence data have also revealed that NFe45079 contains only a single copy of *lpsA*, but all other genes are present in two copies.

### 4.5. Endophyte Genetic Variation within a Single Host Species

The associations of endophytes and hosts represent co-evolving associations as evidenced by the tendency for evolution of the *Epichloë* spp. to track evolution of their hosts [62]. It is interesting to note that sometimes a single host species has symbiotic associations with more than one endophyte species, but each individual plant will only host one endophyte genet. Tall fescue can be symbiotic with *E. coenophiala*, *Epichloë* sp. FaTG-2, FaTG-3 and FaTG-4, and strains representing each of these endophytes have different alkaloid profiles [43,51,60]. *Elymus canadensis* can be symbiotic with *E. elymi* and *E. canadensis*, and each of these endophytes also exhibits chemotype variation [47]. As we develop and refine high throughput methods to explore large host collections more thoroughly, more endophyte variation may be identified within a single host species.

Recently *Bromus laevipes*, a bunchgrass native to California, was found to have independently formed symbiotic associations with three *Epichloë* species, the nonhybrid *E. typhina* subsp. *poae* (designated *Bromus laevipes* Taxonomic Group 1; BlaTG-1), a hybrid designated BlaTG-2, which appears to be phylogenetically similar to *E. cabralii* from *Phleum alpinum*, and another hybrid designated BlaTG-3 [50]. Endophyte diversity within this host collection was identified by PCR-based genotyping of the *EAS*, *LOL*, indole-diterpene/lolitrem (*IDT*/*LTM*) and peramine (*PER*) loci. The BlaTG-3 isolates could be separated into three genotypes (G1, G2, and G3) based on the *EAS* and mating type complement. Isolates considered BlaTG-3 G1 only contained *dmaW*, whereas BlaTG-3, G2, and G3, had different mating-type genes, but shared the same *EAS* gene complement (*EAS^CC^*) in keeping with its ability to produce CC. In contrast, the complete *EAS* gene complement required for ERV production (*EAS^ERP^*) was identified within BlaTG-2, yet ergot alkaloids were not detected in plants with BlaTG-2. RT-PCR expression analyses of the *EAS* genes from BlaTG-2-infected plants indicated they were not expressed. Interestingly, although BlaTG-2 and *E. cabralii* from *Phleum alpinum* are hybrids with the same two ancestral *Epichloë* species, no *EAS* genes are present in characterized *E. cabralii* strains.

Sleepygrass plants growing in the vicinity of Cloudcroft, New Mexico, U.S.A., are renowned for their narcotic effects on livestock because they can have high levels of the ergot alkaloids ergine and EN [63]. It is now clear that two endophyte species can be symbiotic with sleepygrass, *E. funkii* [64] and a so-far undescribed hybrid *Epichloë* species designated AroTG-2 [10]. Each of these endophytes has the *EAS* complement and ability to produce different ergot alkaloids, either CC (*EAS^CC^*) or ergine and EN (*EAS^EN^*), respectively [10]. Phylogenetic analysis of *dmaW* groups the *E. funkii* gene in a clade with *dmaW* of the *E. festucae* clade (Figure 8). In contrast, AroTG-2 has a *dmaW* sequence more similar to that of *E. inebrians* from drunken horse grass than to that of other *Epichloë* species (data not shown), which is in keeping with the similarity of the alkaloids produced by these two endophyte species and their strong stupor-inducing effects on grazing livestock [11,63].

## 5. Synteny and Rearrangements in the *EAS* Loci

### 5.1. Syntenic Regions of the *EAS* Loci

**Figure 10:**
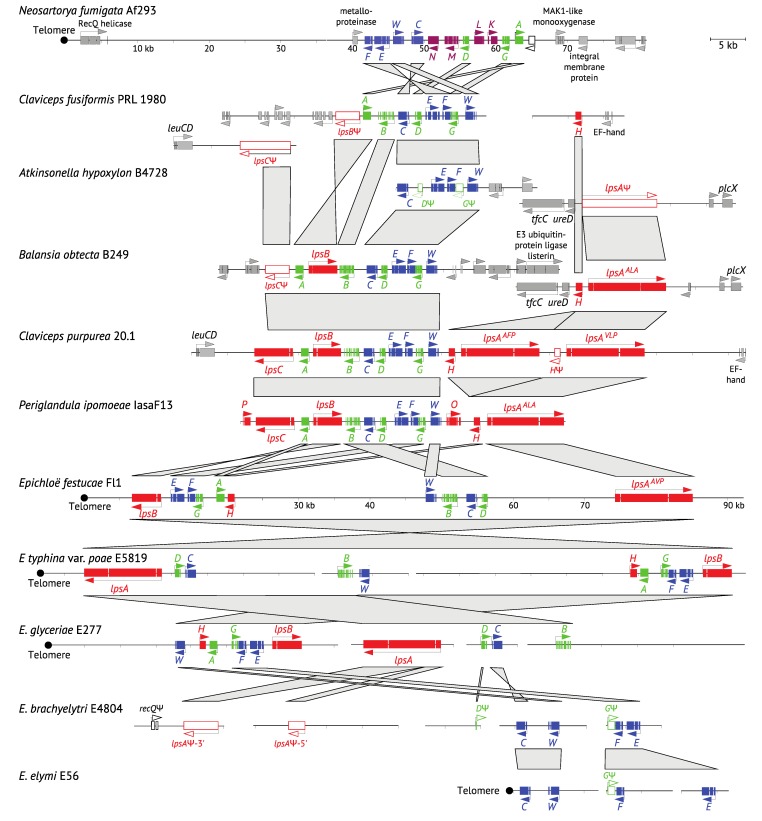
Structures of representative *EAS* loci showing synteny of *EAS* genes between species. Genes are colored to represent the stage of the pathway for the encoded product (see Figure 1 and Figure 2). Pseudogenes are represented by Ψ and white-filled arrows. Gray polygons link orthologous genes and gene blocks but are not meant to imply particular phylogenetic relationships. The *EAS* crown clade includes clusters from *At. hypoxylon*, *B. obtecta*, *C. purpurea*, *C. fusiformis* and *P. ipomoeae*.

Although functional *EAS* genes are always clustered, they are not always in a single cluster, arrangements of the genes are not highly conserved, and locations of the clusters can vary, some being subterminal (near chromosome ends), and others being internal and flanked on both sides by long regions rich in housekeeping genes (Figure 10). With the exception of the *Epichloë* species (discussed below), the degree of divergence in *EAS* gene arrangements, gene contents, and the pathway end-products generally relates to the degree of divergence between species. Thus, it is unsurprising that the *EAS* cluster arrangements and gene contents differ greatly in comparison of *N. fumigata* to the Clavicipitaceae. A surprisingly consistent feature is the arrangement and close linkage of *easE* and *easF* in all except *E. elymi* E56; even their *Ar. benhamiae* orthologues, respectively designated ARB_4648 and ARB_4647, are adjacent but arranged tail to tail [18]. However, *EAS* gene arrangements and orientations are very similar in what we will call the “crown *EAS* clade” (Figure 3): *Metarhizium* spp. (including *Metarhizium acridum*, which is not shown), *P. ipomoeae*, *B. obtecta*, *At. hypoxylon*, *Claviceps* spp. and *E. inebrians*. Differences within the crown *EAS* clade were as follows: (1) an inversion of *lpsB-easA* segment in *C. fusiformis* relative to the others; (2) separation of the *easH-lpsA* segment from others in *B. obtecta*, due to an event that (based on remnant genes) occurred in a common ancestor of *B. obtecta* and *At. hypoxylon*; (3) breakage of the cluster in *E. inebrians* by a telomere introduced immediately downstream of *cloA*; and (4) several gene losses or inactivations that have resulted in changes in ergot alkaloid profiles, as discussed above.

Despite the conserved arrangement of *EAS* genes in the aforementioned crown *EAS* clade, there is very limited synteny of flanking genes (Figure 2), except within the *Claviceps* subclade and, separately, within the *B. obtecta/At. hypoxylon* subclade. The only group of orthologous genes that flanks most members of the crown *EAS* clade is represented in *C. purpurea* 20.1 by AET79176 (GenBank accession number; labeled with asterisks in Figure 2) and is similarly positioned in all except *E. inebrians*, which has its AET79176 orthologue at the opposite end of the cluster*.* With the possible exceptions of *At. hypoxylon* B4728 and *E. festucae* E2368, the AET79176 orthologue was linked to *EAS* in every strain that had one. In B4728, it is also possible that the gene is linked to the *EAS* cluster, but at >75 kb from *easC* (the AET79176 orthologue being near the middle of the 123,479-bp contig00086, which is otherwise very AT-rich and lacks other identifiable genes). So far, there is no putative function or conserved signature domain for AET79176, but it might warrant future investigation for a possible role in the regulation of *EAS* genes or in a biosynthetic role yet to be identified.

### 5.2. Epichloë Species Have More *EAS* Loci Rearrangements

The *Epichloë* species show the greatest variation in cluster organization (Figure 10). Even *EAS^ERP^* clusters, which possess apparently functional forms of 11 *EAS* genes, show extreme rearrangements of gene positions relative to each other and to the telomeres. (The actual linkage and order of the contigs containing *EAS* genes was established for *E. festucae* strains E2368 and Fl1 [24] but could not be determined for other genome sequences when not assembled into scaffolds.) Also highly variable among *Epichloë EAS* clusters was the organization of their extensive, AT-rich, repetitive sequences, which may directly facilitate cluster instability and rearrangements, and in some cases cause partial or entire deletion of genes giving, for example, *EAS^CC^* clusters (Figure 2 and Figure 9) [24]. In addition, within the *Epichloë* species, there is a strong tendency for the *EAS* locus to be retained at the subterminal region, even though the order of genes relative to the telomere (chromosome end) can differ.

Miniature inverted-repeat transposable elements (MITEs) are prevalent in *Epichloë* species [57], and have been identified in the promoters of some *EAS* genes with expansion in the *EAS^CC^* clusters associated with *dmaW* and *easC*. The repetitive AT-rich sequences and prevalence of MITEs are also associated with the gene clusters for biosynthesis of other alkaloid classes; namely, *IDT*/*LTM* (indole-diterpenes) and *LOL* (lolines) [24]. The presence of long transposon-derived repeat blocks seems consistent with the subterminal location of telomere-linked clusters like *EAS* and *IDT*/*LTM*, but the fact that they also feature prominently in the *Epichloë LOL* clusters, which are typically internal with extensive genic regions flanking both ends, suggests that this is a more general feature of alkaloid clusters [24].

### 5.3. The Complex History of *EAS* Loci

**Figure 11:**
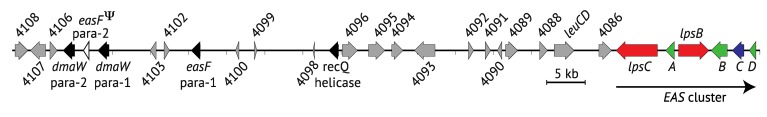
Gene map showing *dmaW* and *easF* paralogues in the region flanking the *EAS* locus from *C. purpurea* strain 20.1. The genes for recQ helicase and paralogues of *dmaW* and *easF* are shown in black, and the genes pertaining to the *EAS* cluster are color-coded based on position of the encoded step within the pathway. For other genes, the locus_tag names (GenBank) are CPUR_04108, CPUR_04107, *etc.*, where only the last four digits are shown. Names of *EAS* genes are abbreviated as in Figure 2.

Although not by any means restricted to subtelomeric and subterminal regions, blocks of transposon-derived repeats are features of these genomic regions in fungi [65,66], and such regions are prone to considerable instability and gene duplication events [67,68]. A subterminal location appears to be the ancestral state of the *EAS* cluster, considering that it is a shared feature of *N. fumigata* and the basal *EAS* clade in Clavicipitaceae; namely, the clade comprised of the majority of *Epichloë EAS* clusters (Figure 3). Thus, increased stability, particularly of the *EAS* core containing early- and mid-pathway genes, seems to be in keeping with the shift from subterminal to internal location in the common ancestor of the crown *EAS* clade. Nevertheless, the differences in flanking housekeeping genes between the *Claviceps* subclade and the *B. obtecta*/*At. hypoxylon* subclade, plus indications of gene duplication in *C. purpurea*, indicate additional complexity in the history of the *EAS* clusters. In *C. purpurea*, the duplication of *lpsA* has enabled its neofunctionalization to greatly enhance the diversity of ergopeptine products (Figure 10). Interestingly, 55-73 kb downstream of *lpsC* in the *C. purpurea* genome are two additional copies of *dmaW* and *easF* (Figure 11), suggesting that gene duplication has been a particularly dynamic evolutionary process in *C. purpurea* in addition to providing an attractive explanation for the fact that most of the known ergopeptin(in)es are reported from this species. Furthermore, the *dmaW* and *easF* duplications are close to a recQ helicase pseudogene, and reflecting their typical locations, recQ helicase genes are also called telomere-linked helicase genes (*TLH*). (For example, a recQ helicase gene is located near the *EAS*-linked telomere of *N. fumigata*, as shown in Figure 2 and Figure 9). In *C. purpurea*, the association of a recQ helicase pseudogene with duplicated *EAS* genes and in the vicinity of the *EAS* cluster suggests more recent evolutionary history associated with a chromosome end than implied in our phylogenetic inferences (Figure 3). Thus, repeated shifts between subterminal and internal locations may have characterized *EAS* clusters in the Clavicipitaceae, and perhaps especially in *C. purpurea* as a driver of ergopeptine diversification.

## 6. Conclusions

Our understanding of ergot alkaloid biosynthesis has greatly increased through genomics and dissection and manipulation of the biochemical pathway. The genetic basis for ergot alkaloid chemotype diversification can be equated to the presence or absence of genes within the *EAS* loci that result in the *EAS* gene complements for, e.g., *EAS^CC^*, *EAS^EC^*, *EAS^FC^*, *EAS^ERP^*, *EAS^EN/ERP^* and *EAS^LAH/ERP^*. Neofunctionalizing changes affecting substrate or product specificity of key enzymes, such as EasA (isomerase *versus* reductase isoforms), CloA and LpsA, also increase pathway diversification.

Phylogenetic analysis of the genes *dmaW*, *easF*, *easC* and *easE*, which are common to all ergot alkaloid producers, has provided insight into the gene gains and losses that drive chemotypic diversification. In addition, phylogenetic relationships of the *EAS* genes are not congruent with those of housekeeping gene (e.g., *tefA*) phylogeny, as the majority of the *Epichloë* *EAS* genes (excluding those from *E. inebrians*) do not subtend the *Claviceps* clade and may represent paralogous *EAS* clusters. What also stands out among the sequenced *Epichloë* strains is the large amount of *EAS*-associated AT-rich repetitive sequences, in comparison to the *EAS* loci from the other Clavicipitaceae and *N. fumigata*. These repetitive sequences, as well as subterminal locations, have likely impacted the *Epichloë* *EAS* gene content and organization. We have also presented evidence above (Section 5) that subterminal locations are associated with gene duplication and neofunctionalization even in the evolution of the currently internal *EAS* cluster of *C. purpurea*.

Unique to the *Epichloë* species is the tendency for hybrid formation, and in this process one or more of the ancestors may contribute *EAS* clusters. For some hybrid strains, the individual *EAS* contribution is incomplete, yet if two copies are present, each *EAS* cluster can functionally complement the genetic deficiencies of the other clusters.

Genome sequence comparisons between species and strains show that the *EAS* loci can vary considerably based on distribution, gene content, gene order, and associated repeat content. Variations identified across multiple *EAS* loci are all in keeping with the overall natural chemotype diversity that has been identified within the Clavicipitaceae and the Trichocomaceae, and likely provides important selective advantages for many of the species in these families.
